# Genetic parameters via predictivity in large populations under strong genomic selection

**DOI:** 10.1186/s12711-026-01054-9

**Published:** 2026-06-07

**Authors:** Gopal Gowane, Jorge Hidalgo, Mary Kate Hollifield, Ignacy Misztal, Daniela Lourenco

**Affiliations:** 1 Department of Animal and Dairy Science, University of Georgia, Athens, GA 30602 USA; 2 Animal Genetics and Breeding Division, ICAR-National Dairy Research Institute, Karnal, 132001 India

## Abstract

**Background:**

Estimation of accurate genetic parameters require using all data that were available upon selection. Existing methods for parameter estimation become impractical with large genomic data, and estimation becomes complex if parameters vary over time. This study aimed to test a new method called ‘GPP’ (Genetic parameters via predictivity), which combines within and across traits predictivity formulas with a deterministic formula to predict the accuracy of genomic breeding values. The GPP method can handle any data size and can estimate parameters across time slices.

**Results:**

We tested GPP using simulated datasets with negatively and positively correlated traits: primary production (h^2^ = 0.40), secondary production (h^2^ = 0.10) and a fitness (h^2^ = 0.10) trait. Genomic selection was based on the primary production trait Two scenarios included either 5000 (A) or 100,000 (B) genotyped animals per generation for 10 generations. Across the two scenarios, GPP estimates closely matched the realized values, with a slight, variable bias. Using incorrect variances to compute GEBV prior to GPP had little impact on estimates. With the smaller data sets, estimates by REML using only one generation were highly variable, and estimates by GREML had lower standard errors. For large data, REML estimates were slightly biased. Genetic correlations obtained with GPP were non-symmetric, meaning they varied depending on which trait adjusted phenotype was used as a benchmark in the formula. We observed lower bias when predictivity involved adjusted phenotypes for the secondary trait. The GPP method performed equally well across both positively and negatively correlated traits, as well as for weak and strong genetic correlation scenarios, in both small and large datasets. Computations took 55 min and 11 s for a one-time slice run of GPP in the large dataset (scenario B). GPP had an approximately linear cost with the number of genotyped animals.

**Conclusions:**

Genetic Parameters via predictivity is a fast, flexible, and accurate approach for estimating dynamic genetic parameters for positively or negatively correlated traits and also for weak and strong genetic correlation scenarios across time slices in large datasets, subject to genomic evaluation, where predictivity computation is feasible.

**Supplementary Information:**

The online version contains supplementary material available at https://doi.org/10.1186/s12711-026-01054-9.

## Introduction

Genetic parameter estimates are not constant over time. In fact, they change with selection due to changing allele frequencies over time (i.e., the Bulmer effect; [1]) and drift [2]. Other reasons causing genetic parameters to change are changing trait definition [3], resource allocation shift [4], nonlinearity among traits [5], and nonadditive effects [6]. Intensively using genomic selection in livestock may further accelerate the rate of changes in genetic parameters [7]. This occurs not only for the trait under direct selection but also for the correlated traits not considered for selection due to differential resource allocation [4, 8, 9] if the selection intensity is very high. Fitness, disease resistance, and heat tolerance are some traits that have been negatively affected due to an increased negative correlation between them and the traits under selection [10]. Misztal and Lourenco [7] presented informal reports that strong genomic selection on production traits has accelerated the negative effect on fitness traits. Hidalgo et al. [11] showed that the h^2^ for a growth trait in pigs halved, and its correlation with a fitness trait became 50% more negative after a few years post-genomic selection.

Genomic selection has become the norm in animal breeding and genetics, so much so that the number of genotyped animals has quickly increased in the last decade. As of March 2025, the number of genotyped animals in the US Dairy database surpassed 10 million (https://uscdcb.com/database-stats/), and this number is around 2 million for the American Angus Association (Pedro Ramos, Angus Genetics Inc., personal communication). Estimating genetic parameters for such large genomic datasets using traditional methods like average information (AI) or expectation maximization (EM) restricted maximum likelihood (REML; [12]) and Bayesian methods via Gibbs Sampling (GIBBS; [13]) is almost impossible as no available algorithms and software can afford the time and cost associated with the estimation. Although REML and GIBBS have good theoretical properties, adding genomic information brings several challenges as the mixed model equations become dense [14]. Using the algorithm for proven and young (APY; [15]) in single-step genomic BLUP (ssGBLUP) can alleviate the computational burden of obtaining the inverse of the genomic relationship matrix (G^− 1^) and make the system much sparser. However, this works well for genomic EBV (GEBV) estimation, not for variance component estimation (VCE). Junqueira et al. [16] observed no savings in computing resources when using APY G^− 1^ for VCE. The authors stated that although APY G^− 1^ was very sparse, the inverse of the pedigree relationship matrix among genotyped animals was still dense. Looking at alternative methods, Hollifield et al. [17] showed it is feasible to apply method R [18] to estimate heritability in large genomic datasets; however, it cannot be used for multiple correlated traits. Using a grid search for variance components that maximize predictivity [19], Hollifield et al. [17] observed that although any data size can be used, the method is not a good estimator of trait heritabilities, as there were minimal differences between the predictivity for each heritability tested, and it cannot work with correlated traits.

To avoid bias in VCE, all sources of information on which the selection was based should be included in the model [20]. Under genomic selection, REML and GIBBS become biased because they do not account for genomic information. The ssGBLUP method is resistant to bias in breeding value estimation due to selection because all the information is considered jointly. Cesarani et al. [21] showed that extending the single-step methodology to VCE (i.e., ssGREML) provided unbiased values because all available phenotypes, pedigree, and genotypes are considered together in the VCE process. However, as discussed before, REML- and GIBBS-based methods are not feasible with many genotyped animals. Aiming to accommodate all the genomic information available, Misztal and Gowane [22] developed a method to estimate heritability, genetic correlations, and their standard errors. This method combines formulas for predictivity [19] within and across traits and a deterministic formula to predict the accuracy of genomic breeding values [23]. We will refer to this method [22] as ‘GPP’, which stands for Genetic Parameters via Predictivity. GPP is a promising method as it can potentially be applied to any data size and can estimate genetic parameters by time slices.

When estimating variance components and genetic parameters using all available generations of data, the results reflect the state of the base population. However, to investigate temporal changes in genetic parameters as a consequence of genomic selection without referring back to the base population, we need a method that works with time slices. This study used the GPP method to investigate how genetic parameters for positively and negatively correlated traits of varying strength changed over time when only one production trait was under strong genomic selection. We used small and large simulated datasets and compared results from GPP with those from genomic and pedigree REML when the data size allowed. Together, these objectives allowed us to evaluate the robustness and applicability of the GPP method for estimating genetic parameters under conditions of strong directional genomic selection.

## Methods

### Data simulation

The genotypic and phenotypic data were simulated using the AlphaSimR package [24] in R (v4.4.0). The genome of the historical population was created using the Markovian Coalescent Simulator (MaCS) [25] implemented in the runMacs function of AlphaSimR package. A total of 100,000 founder haplotypes were simulated across 30 chromosomes, each containing 2000 segregating sites (60,000 segregating loci genome-wide), using the built-in cattle demographic model (species = “CATTLE”), which incorporates historical recombination and mutation processes under a species-specific coalescent framework [26]. Animals in the base population were randomly selected and the number of males (Nm) and females (Nf) at current generations was managed to create an effective population size of 50 based on Wright’s formula $\:{N}_{e}$ = 4NmNf/(Nm + Nf). In total, 49,950 biallelic markers (SNP) (1665 per chromosome) were sampled from the segregating sites. SNP and QTL positions were mutually exclusive, and no overlap between marker and QTL loci was allowed. All SNPs and QTL were segregating in the founder population, as they were sampled from polymorphic sites generated during the coalescent simulation. No additional mutation was introduced after the founder generation; loci were allowed to segregate, drift, or reach fixation during subsequent generations of selection. From the segregating loci generated by the coalescent simulation, 3000 quantitative trait loci (QTL; 100 per chromosome) were randomly sampled to define two additive traits.

Two trait phenotypes were simulated for each animal. Trait 1 mimicked a production trait and had a heritability of 0.4, whereas trait 2 had a heritability of 0.1, with no genetic correlation between them. We then created trait 3, which was the “real” fitness trait and had the same variance as trait 2 but a declining genetic correlation with the production trait over generations. Trait 3, which mimicked a ‘fitness trait’, was created using the following equation:

$$\:{u}_{3}^{j}={\upalpha\:}({u}_{2}^{j}-\beta\:{u}_{1}^{j}(\overline{{u}_{1}^{j}-{u}_{1}^{0}}))$$

where $\:{u}_{i}^{j}$ is the breeding value for trait *i* in generation *j*. The parameter ‘$\:\beta\:$’ controlled the change in the genetic correlation every generation and parameter ‘$\:{\upalpha\:}$’ kept the variance for trait 3 the same as that for trait 2; therefore,

$$\:var\left({u}_{3}^{j}\right)=var\left({u}_{2}^{j}\right)$$

Trait 4, which was a secondary production trait, had the same variance as trait 2 and increasing genetic correlation with the primary production trait over generations. Trait 4 was created using the following equation:

$$\:{u}_{4}^{j}={\upalpha\:}({u}_{2}^{j}+\beta\:{u}_{1}^{j}(\overline{{u}_{1}^{j}-{u}_{1}^{0}}))$$

where all terms were as defined above. We used $\:{\upalpha\:}$ to keep the same variance for trait 4 as in trait 2,

$$\:var\left({u}_{4}^{j}\right)=var\left({u}_{2}^{j}\right)$$

Following the previous conditions, we simulated two recent populations with varying numbers of animals per generation: 5000 (scenario A) and 100,000 (scenario B). For that, we followed the subsequent steps: From the founder population, 13 males and 2500 females (scenario A) or 50,000 females (scenario B) were selected randomly to produce generation 1 with equal probability of having male or female progeny. Parents were selected based on their genomic estimated breeding values (GEBV). For the GEBV computation, AlphaSimR externally called BLUP90IOD3 from the BLUPF90 software suite [27] every generation. 50% of the females were replaced each generation. The simulation was replicated five times in both scenarios.

### Genetic parameters estimation using established methods

We estimated the genetic parameters in time slices that contained data up to three overlapping generations. Therefore, eight time slices were established: G1-3, G2-4, G3-5, G4-6, G5-7, G6-8, G7-9, G8-10. For the small dataset (scenario A) genetic parameters were estimated under REML and GREML using BLUPF90+ [15, 28], for each generation separately and also for sets of two and three generations together for the eight time slices. For the large dataset (scenario B), genomic REML (GREML) estimation was not possible; therefore, pedigree-based REML estimates for one generation were obtained [15, 28].

### Genetic parameters estimation via predictivity

Misztal and Gowane [22] derived an equation to compute heritability and its standard error using a formula for predictivity [19] and a deterministic formula [23] to predict the accuracy of genomic breeding values:

1 $$\:\widehat{{h}^{2}}=\frac{{c}^{2}+\sqrt{{c}^{4}+4{c}^{2}{M}_{e}/N}}{2}$$

where *N* is the number of animals in the reference group; $\:c\:=\:corr(\mathbf{y}-\mathbf{X}\mathbf{b},\widehat{\mathbf{u}})$, which is the correlation between adjusted phenotypes and estimated breeding values (i.e., predictivity) for a focal group (i.e., validation animals); $\:{M}_{e}$ is the number of independent chromosome segments.

The standard error for heritability was obtained as given in [22]:

2 $$\:SE(\widehat{{h}^{2})}=1.5c/\sqrt{n}$$

where * n* is the number of animals in the focal group.

In each time slice, the first two generations were used to predict the genetic parameters for the last generation (e.g., G1-3: generations 1 and 2 were the reference group, whereas generation 3 was the focal group). The number of animals in the reference (focal) group was *N* = 10,000 (* n*=5000) for scenario A and *N* = 200,000 (* n*=100,000) for scenario B. For these scenarios, $\:{M}_{e}$ was computed as the number of largest eigenvalues explaining 98% (eig98) of the variance in G [29] in each generation of focal animals (Additional file 1 Table S1). For scenario A, the average estimates of eig98 varied from 3475 in generation 1 to 2888 in generation 10, whereas in scenario B, they varied from 8801 in generation 1 to 7142 in generation 10.

To estimate genetic correlations between traits * i* and * j*, Misztal and Gowane [22] derived the following equation, where * i* and * j* can be switched to compute $\:{Cor}_{ij}$ or $\:{Cor}_{ji}$:

3 $$\:{Cor}_{ij}=Cor({\mathbf{y}}_{\mathrm{i}}-{\mathbf{X}\mathbf{b}}_{\mathrm{i}},\widehat{{\mathbf{u}}_{\mathrm{j}}})/{h}_{i}{acc}_{j}$$

where, * h* is the square root of the heritability as obtained in (1) for either trait * i* or * j.* The * acc* is the estimate of accuracy from Legarra et al. [19], which is: * c/h*. The standard errors for genetic correlation can be obtained as:

4 $$\:SE\left({Cor}_{ij}\right)\approx\:1/\left({h}_{i}{acc}_{j}\sqrt{n}\right)$$

where * i* and * j* can be switched to compute $\:{SE(Cor}_{ij})$ or $\:{SE(Cor}_{ji})$.

The realized accuracy of predicting GEBV was obtained as correlation between true breeding values (TBV) and GEBV.

In a nutshell, the pipeline to use GPP for estimating genetic parameters is as follows:

1. Run a genomic evaluation to compute GEBV using pedigree and genotypes for the reference and focal groups and phenotypes only for the reference group; use known heritabilities and assume 0 covariance among traits.
2. Run a second genomic evaluation with phenotypes, pedigree, and genotypes for reference and focal groups; use known heritabilities and assume 0 covariance among traits. Adjust phenotypes for fixed effects. Only adjusted phenotypes for the focal group are used in the next step.
3. Calculate predictivity.
4. Calculate heritabilities and SE using Eqs. (1) and (2).
5. Calculate genetic correlations and SE using Eqs. (3) and (4).
6. Repeat steps 1 to 5 for each time slice.

In our study, we used the BLUPF90 software suite [27], performing BLUP for small data with BLUPF90 + and for large data with BLUP90IOD3. Phenotypes were adjusted using PREDICTF90.

## Results and discussion

### Genetic trends for the simulated traits

**Fig. 1:**
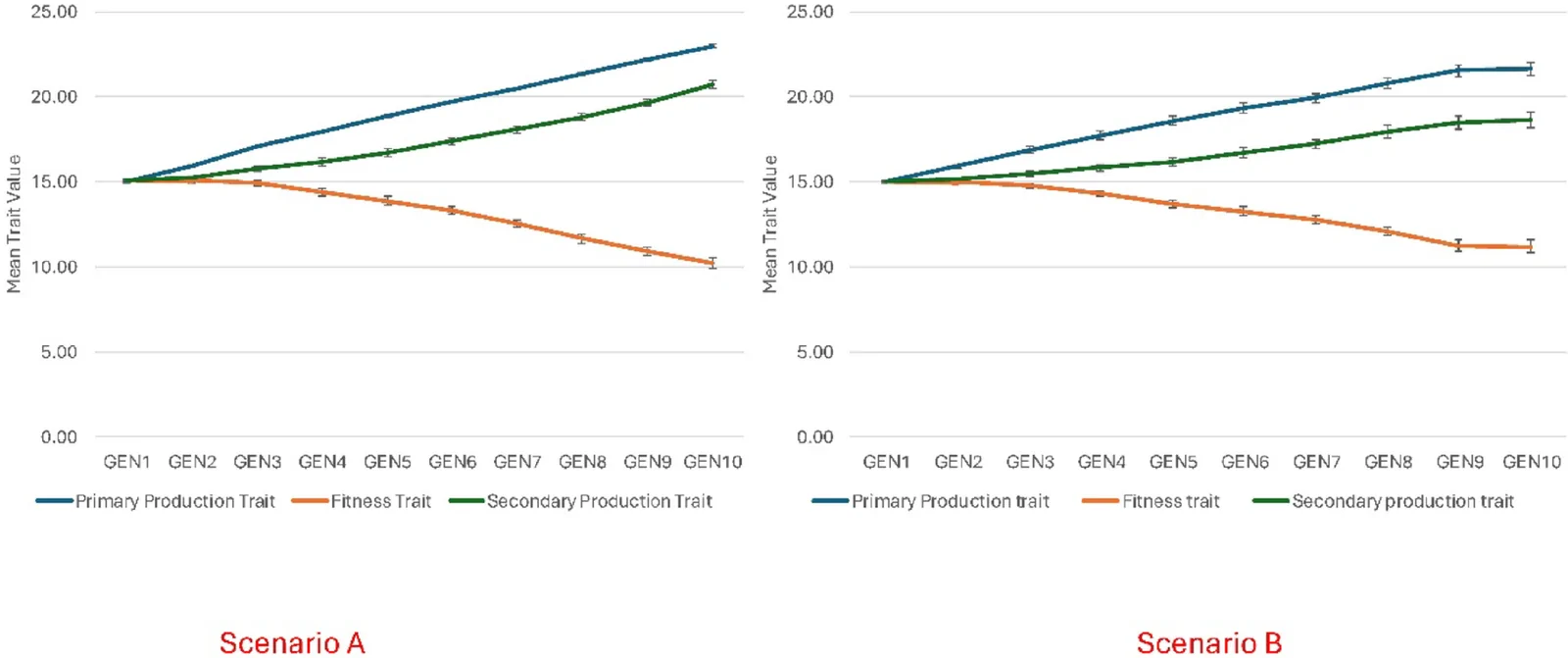
Genetic trends for production and fitness traits for large and small datasets. Standard errors (SE) are SE of replicates

The intensive genomic selection on the primary production trait resulted in increased genetic gains for this trait and a deterioration in fitness due to the decreasing correlation with the primary production trait (Fig. 1). This was true for small and large datasets. Although no direct selection was applied to fitness, the trait was composite and included a greater fraction of the production trait with each generation. Additionally, even though the secondary production trait was not under direct selection, there was an improvement in this trait due to increasing correlation with the primary production trait.

In the large-data scenario (B), the relative gain in the phenotypic mean was modest. Response is a function of heritability and selection differential. Because a larger number of females per generation was selected for mating in scenario B, the selection differential was smaller, contributing to the reduced response. The larger number of selected females likely diluted the genetic contribution of each selected male, further limiting short-term gain. Recombination may have played a role in large populations by slowing the loss of additive genetic variance over generations. In AlphaSimR, new haplotypes are generated by explicitly modeling genetic recombination during meiosis in diploid species [30].

### GPP for small datasets

**Fig. 2:**
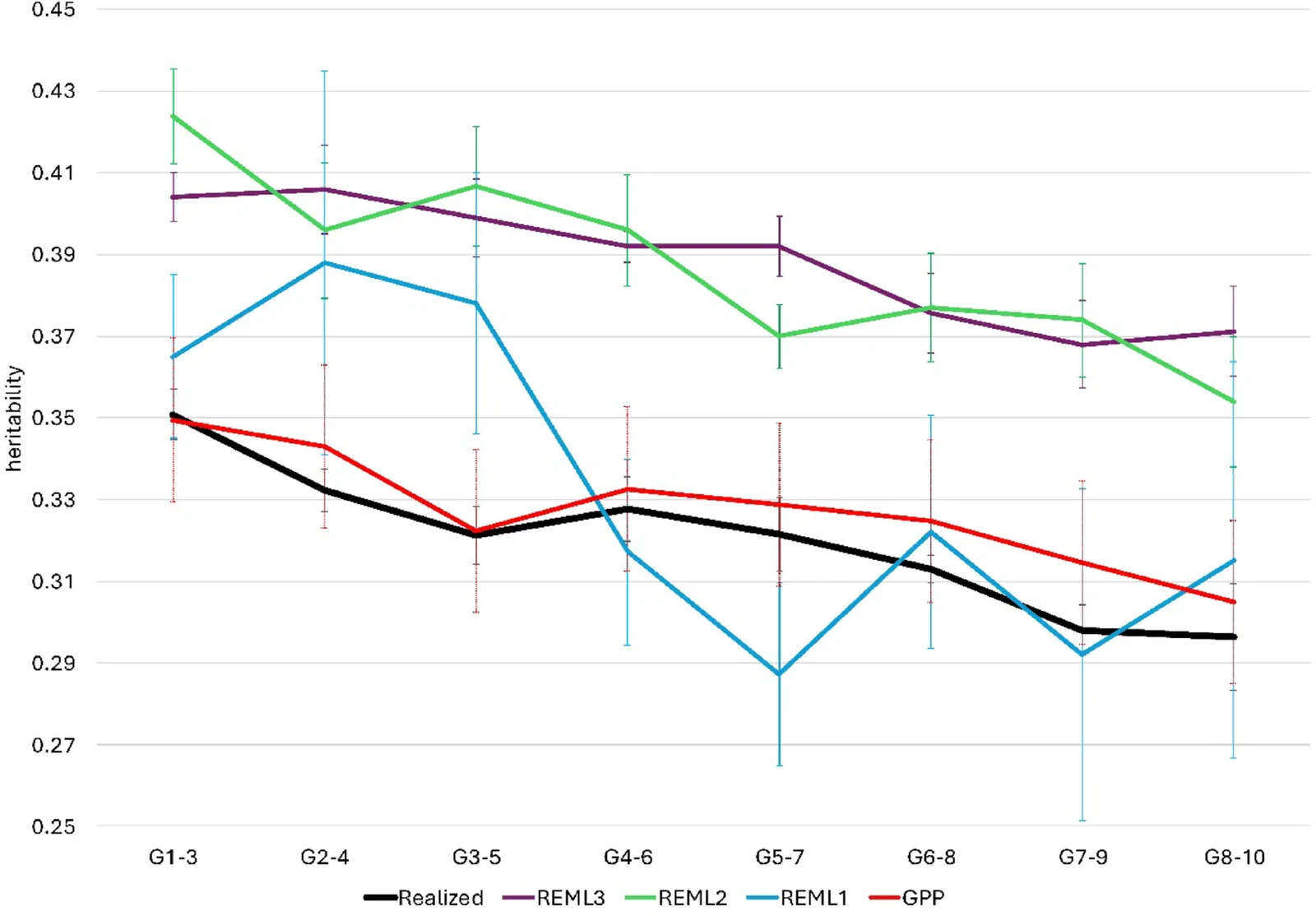
Realized and estimated heritability over generations for production in the small dataset. Estimates are by pedigree-based REML using 1 to 3 generations of phenotypes and by genetic parameters via predictivity (GPP). Standard errors (SE) are SE of replicates

Figure 2 presents realized and estimated heritabilities via REML and GPP for production as a function of generation number. The GPP estimates for a given generation used adjusted phenotypes from that generation, and GEBV obtained from observations of two previous generations. Three estimates are available by pedigree-based REML: using a single generation, two generations (current and prior), and three generations (current and two prior) of data. The realized heritability declined from 0.35 to 0.30 due to the Bulmer effect. The estimate by GPP closely followed the realized heritability. Estimates by pedigree-based REML were mostly above the realized values, with the highest values with three generations of data and the highest fluctuations with a single generation of data. These results are as expected because the standard errors decrease with data size, and pedigree-based REML refers to the base generation.

**Fig. 3:**
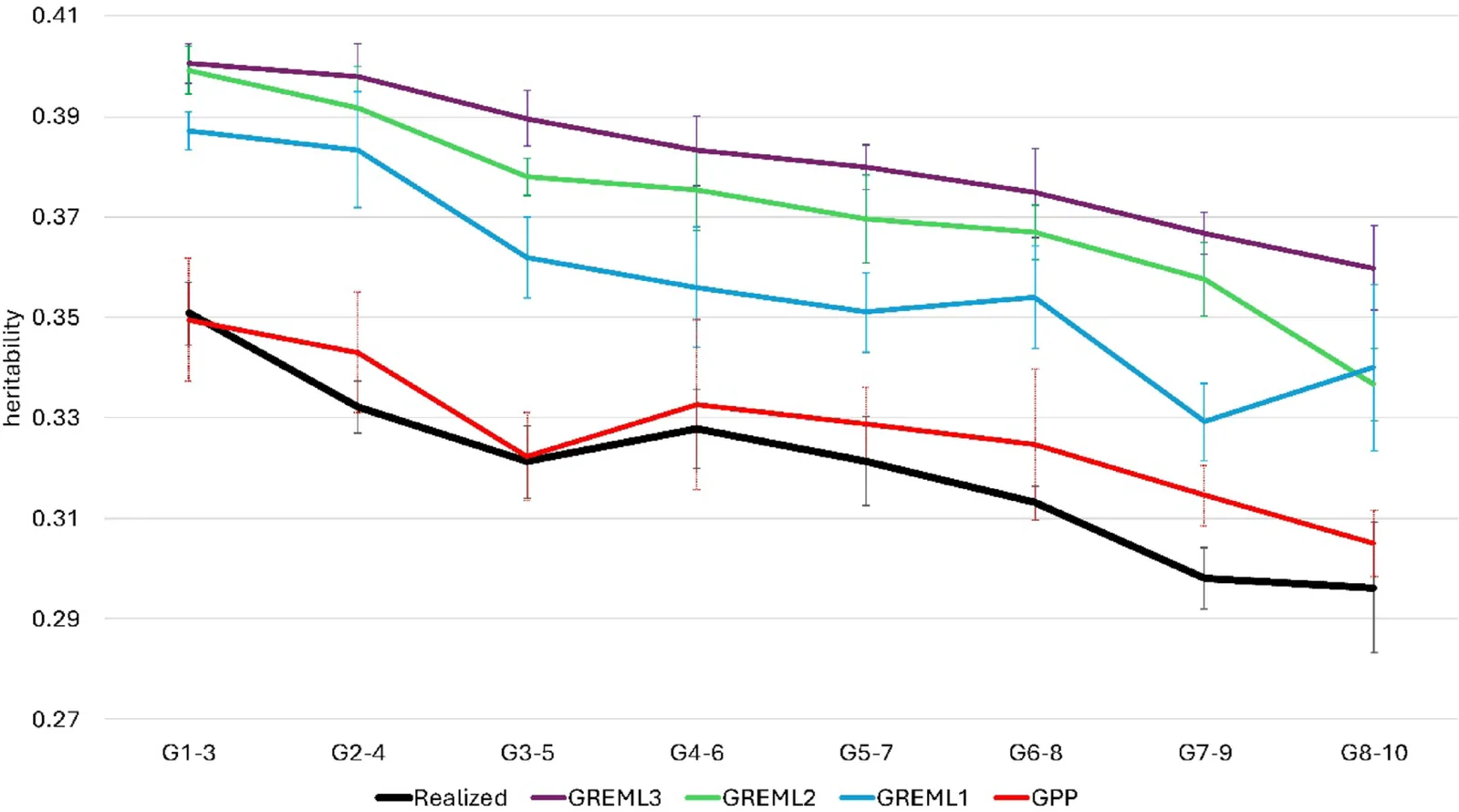
Realized and estimated heritability over generations for production in the small dataset. Estimates are by genomic REML (GREML) using 1 to 3 generations of phenotypes and genetic parameters via predictivity (GPP). Standard errors (SE) are SE of replicates

Figure 3 presents heritabilities as in Fig. 2, except that pedigree-based REML was replaced by GREML. On average, the estimates were similar to pedigree-based REML; however, with less fluctuations. In a population not yet undergoing genomic selection, estimates by REML ignoring or using genomic information were similar, but with lower standard error [31]. Estimates by pedigree-based REML and GREML differ depending on details. In general, pedigree-based REML estimates refer to the base population. In real situations, the base population may not be clear. For example, if pedigrees are much deeper than the phenotypes, the estimates may refer to a generation between the oldest phenotypes and the oldest pedigree. Missing pedigrees across generation have an impact too as they create multiple base populations. In GREML, the estimate depends on how the genomic relationship matrix is constructed. If **G** is constructed as per VanRaden [32], parameters are dependent on SNP allele frequencies. Additionally, the similarity between **A** and **G** requires using the base allele frequencies [32]. If using single-step GREML (ssGREML), **G** is scaled for similarity with **A** [33–35]. In this study, we used average allele frequencies computed based on the simulated genotyped population; therefore, the estimates would be averages across generations. In pedigree-based REML and GREML, the overestimation increased when adding more generations, probably due to the use of current instead of base-population allele frequencies.

**Fig. 4:**
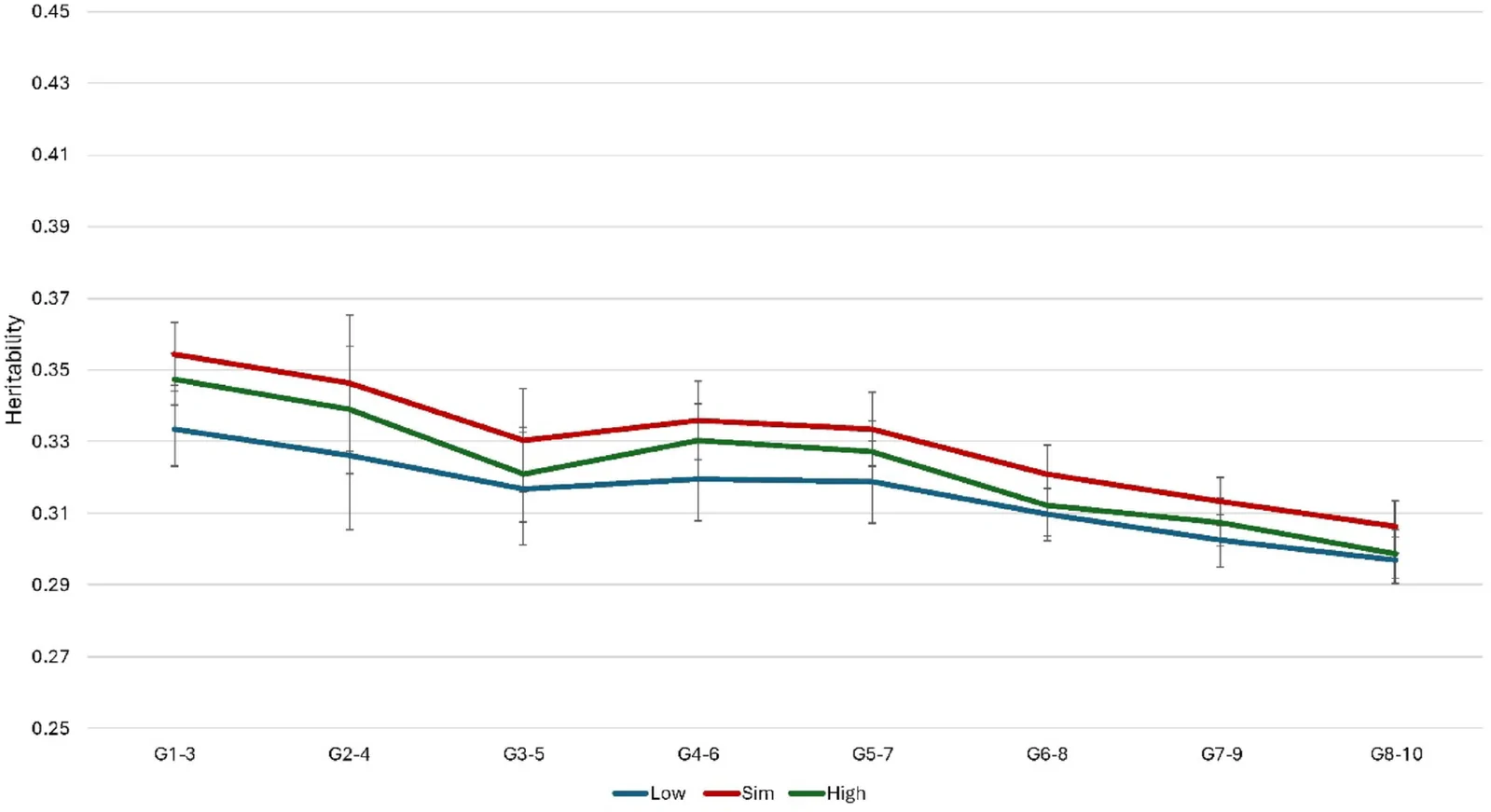
Estimated heritability over generations for production in the small dataset by genetic parameters via predictivity (GPP) when GEBV were computed with the additive variance as simulated (Sim), reduced by 50% (Low) and doubled (High). Standard errors (SE) are SE of replicates

Estimates by GPP use GEBV, which are calculated with parameters that may be very different from the true ones, as they are usually based on pedigree information from a subset of the population due to computational limitations. Subsequently, the GPP procedure could be iterative to ensure accurate estimates. To investigate the impact of different parameters when estimating GEBV used in GPP, we used three sets of values: simulated (Sim), halved (Low) and doubled (High) additive genetic variance. As seen in Fig. 4, using different parameters when computing GEBV had a small impact on the heritability by GPP. Although values slightly differed, the standard error bars overlapped. This is true because predictivity is a correlation, and the impact of parameters is similar in the numerator (covariance) and denominator (variances), leading mainly to a cancellation. Thus, it can be expected that, iterations using changing variances to compute GEBV for GPP will not cause considerable changes and, therefore, can be avoided.

**Fig. 5:**
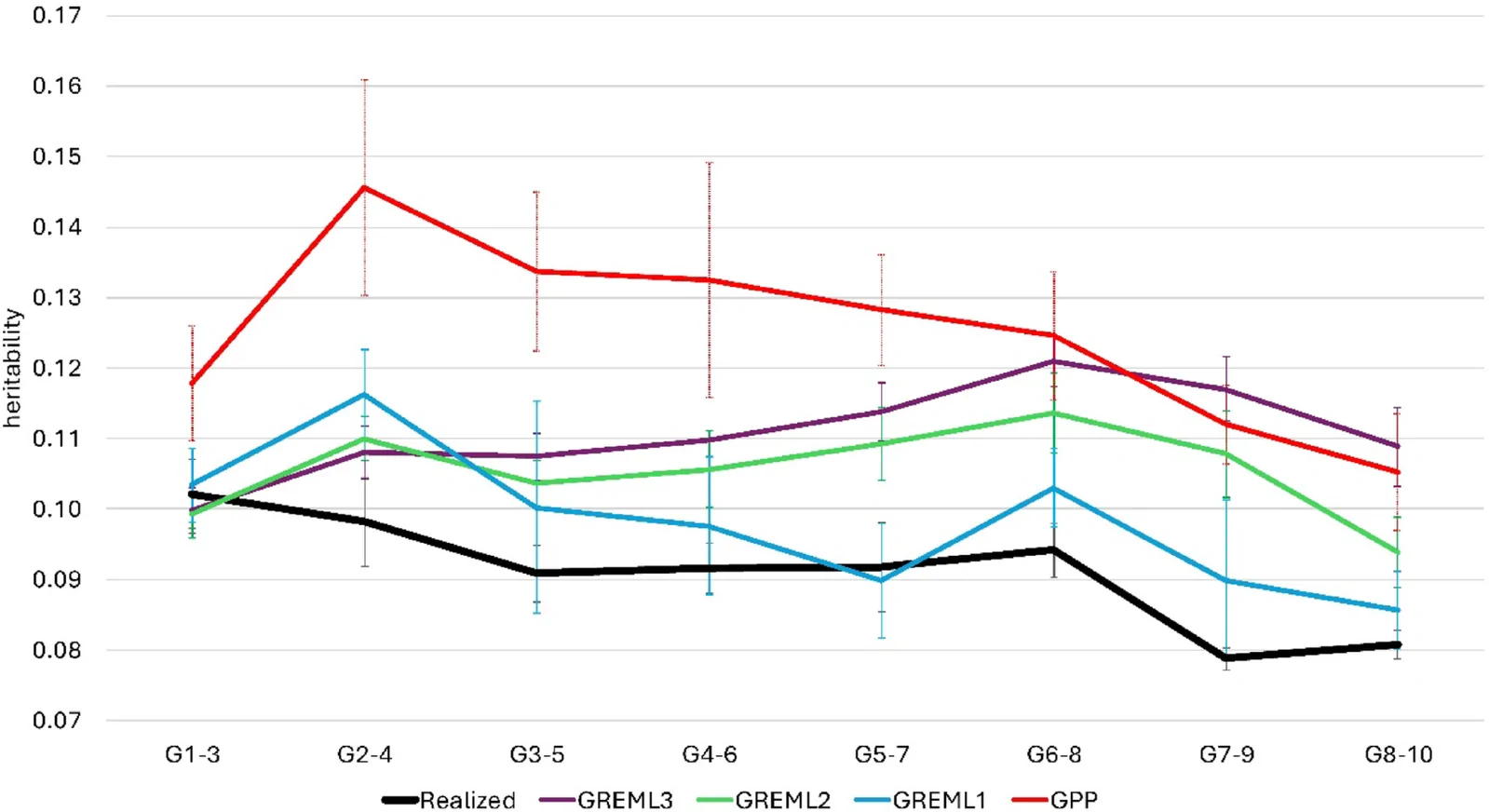
Realized and estimated heritability over generations for fitness in the small dataset. Estimates are by Genomic REML using 1 to 3 generations of phenotypes and by genetic parameters via predictivity (GPP). Standard errors (SE) are SE of replicates

Figure 5 is equivalent to Fig. 3, but for the fitness trait. Contrary to the previous results, GREML estimates from a single generation were relatively closer to the realized values than those from two or three generations. Also, heritability estimates based on GPP were inflated. Overestimation in GREML, especially with more than one generation, may have been due to the use of current instead of base-population allele frequencies. This is evident in Fig. 5, where estimates in earlier time slices are better across GREML scenarios when allele frequency is close to the base population frequency. The fitness trait included an increasing fraction of the production trait each generation; hence, the change in genetic variance was not solely due to selection for the primary production trait. Subsequently, a model of analysis that assumes constant correlation between traits across generations is inappropriate, and analyses with a single generation can be less biased. For GPP, inflation could be due to the reduced applicability of the equation provided by Daetwyler et al. [23] in small data sets and low-heritability traits. In general, GEBV can be decomposed into several components, including the parent average, contributions due to records and progeny, and a genomic component adjusted for the fraction of parent average already present in **G** [36]. The equation of Daetwyler et al. [23] accounts only for the last component, which is dominant in analyses with a large number of genotyped animals.

**Fig. 6:**
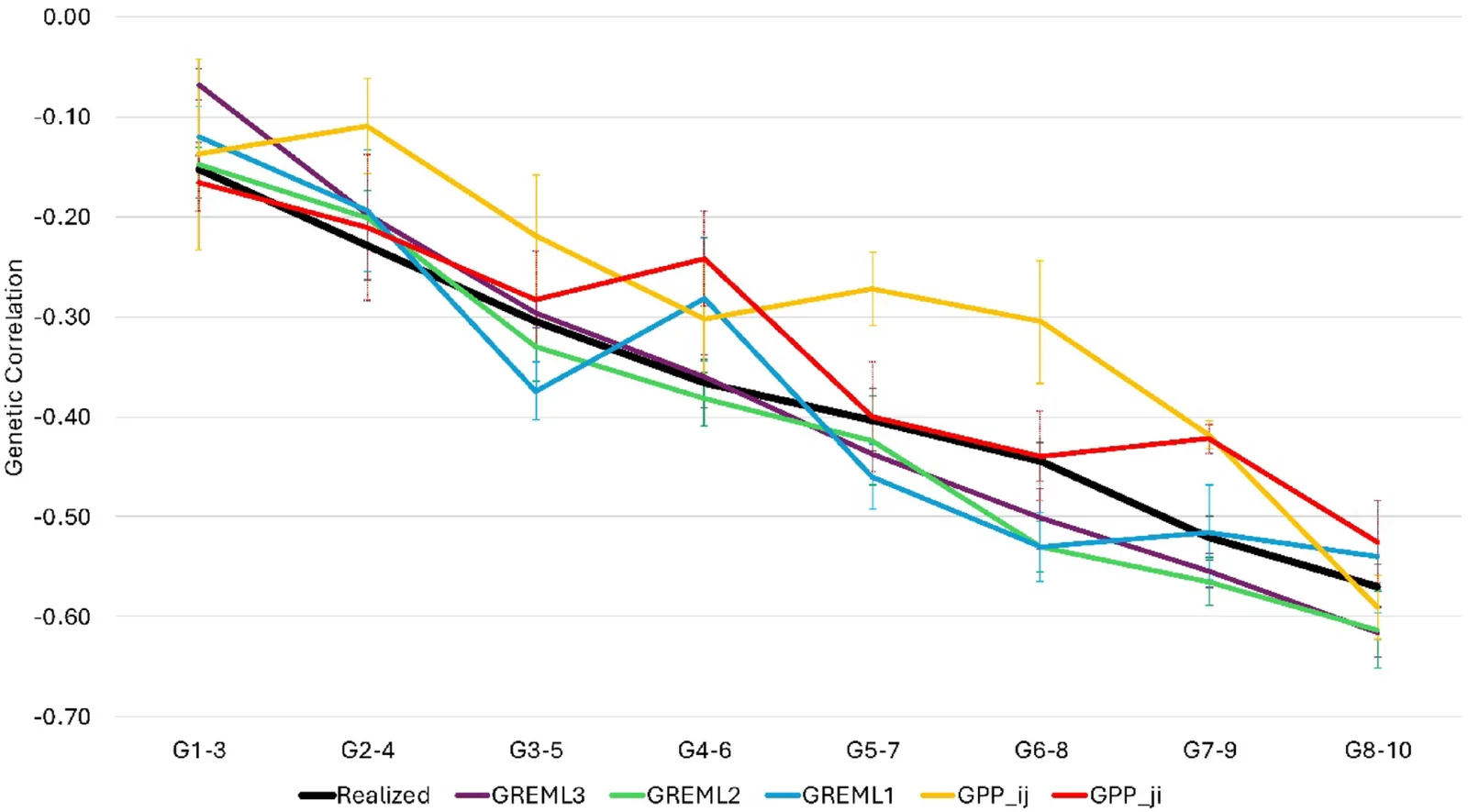
Realized and estimated genetic correlations between fitness and production over generations for the small dataset. Estimates are by Genomic REML (GREML) using 1 to 3 generations of phenotypes, and by genetic parameters via predictivity (GPP). The latter considered predictivity of production based on fitness (Gpp_ij_) and fitness based on production (Gpp_ji_). Standard errors (SE) are SE of replicates

Figure 6 shows genetic correlations between production and fitness over generations for different methods. The realized genetic correlation declined from 0 in generation 1 to − 0.56 in generation 10. As in estimates of heritability for fitness, the genetic correlations estimated by GREML using a single generation are closer to the realized values than those estimated using more generations. GPP estimates were higher and closer to zero than real values due to an artifact of inflated heritability for fitness obtained by GPP. If the higher estimate is due to limited information available for the low-heritability trait, the issue will likely be reduced or eliminated with larger datasets. The correlation strength (weak in earlier time slices and strong in later time slices) did not affect the GPP estimate, which remained underestimated across all time slices.

GPP provides two estimates of each genetic correlations. In our case, one is based on predicting adjusted phenotypes for production based on GEBV for fitness using two previous generations of phenotypes for the latter trait. The second is based on predicting adjusted phenotypes for fitness based on GEBV for production using two previous generations of phenotypes for production. Since fitness changes its composition over generations, the first estimate will be based on correlations in older generations, resulting in a lag in estimates, and the second will utilize the current phenotype without a lag. This can be confirmed in Fig. 6.

**Fig. 7:**
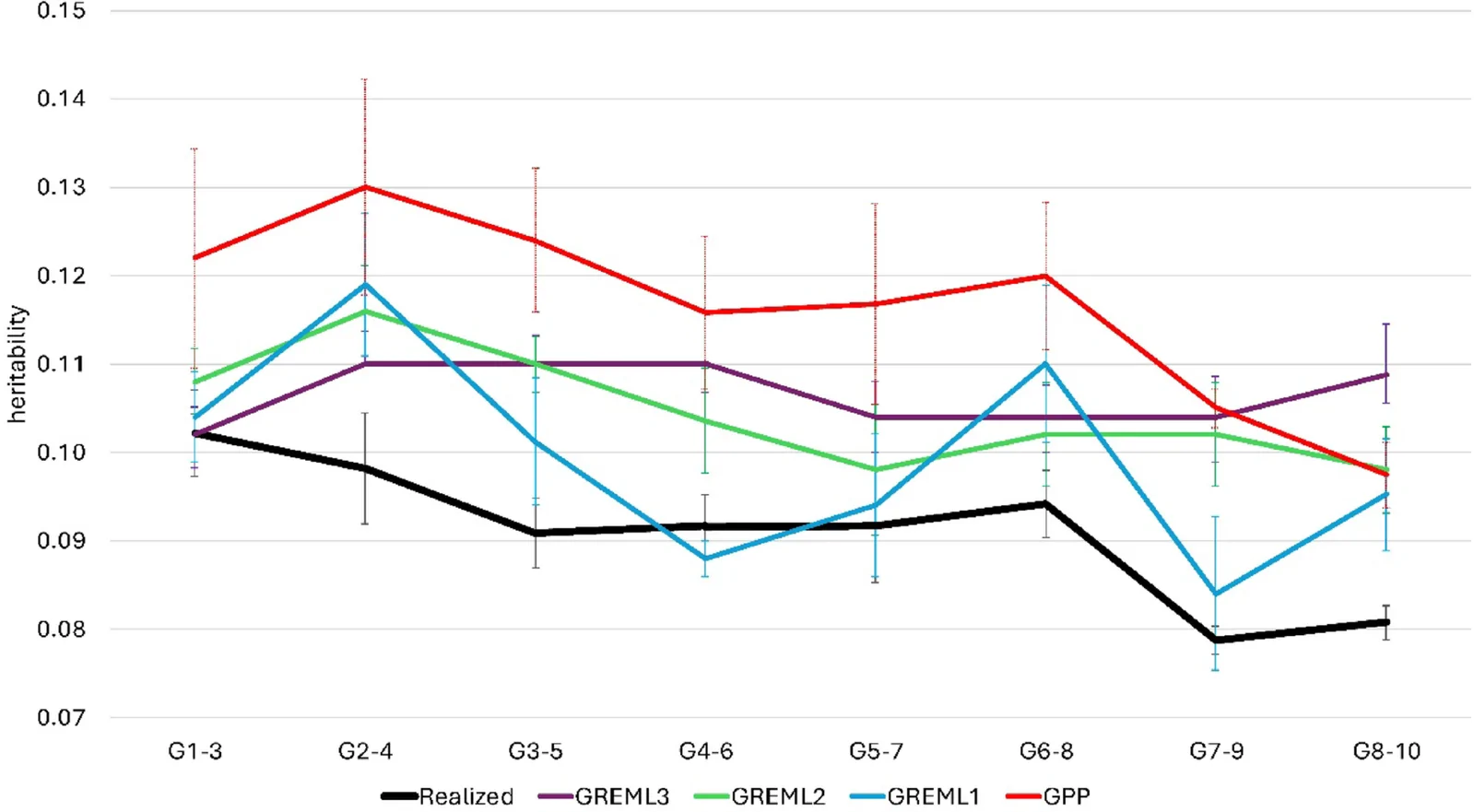
Realized and estimated heritability over generations for secondary production trait in the small dataset. Estimates are by Genomic REML using 1 to 3 generations of phenotypes and by genetic parameters via predictivity (GPP). Standard errors (SE) are SE of replicates

The heritability estimates for the secondary production trait that had a positive genetic correlation with the primary production trait were assessed over the time slices. Similar to the estimates from the fitness trait, GREML estimates using a single generation were more similar to the realized values than those from two and three generations (Fig. 7). Heritabilities by GPP were inflated. The composite trait included an increasing fraction of the production trait every generation. Subsequently, a model of analysis that assumes constant correlation between traits is improper, and analyses with a single generation can be less biased. For GPP, the inflation could be due to the reduced applicability of the equation provided by Daetwyler et al. [23] with small data sets and low heritability traits.

**Fig. 8:**
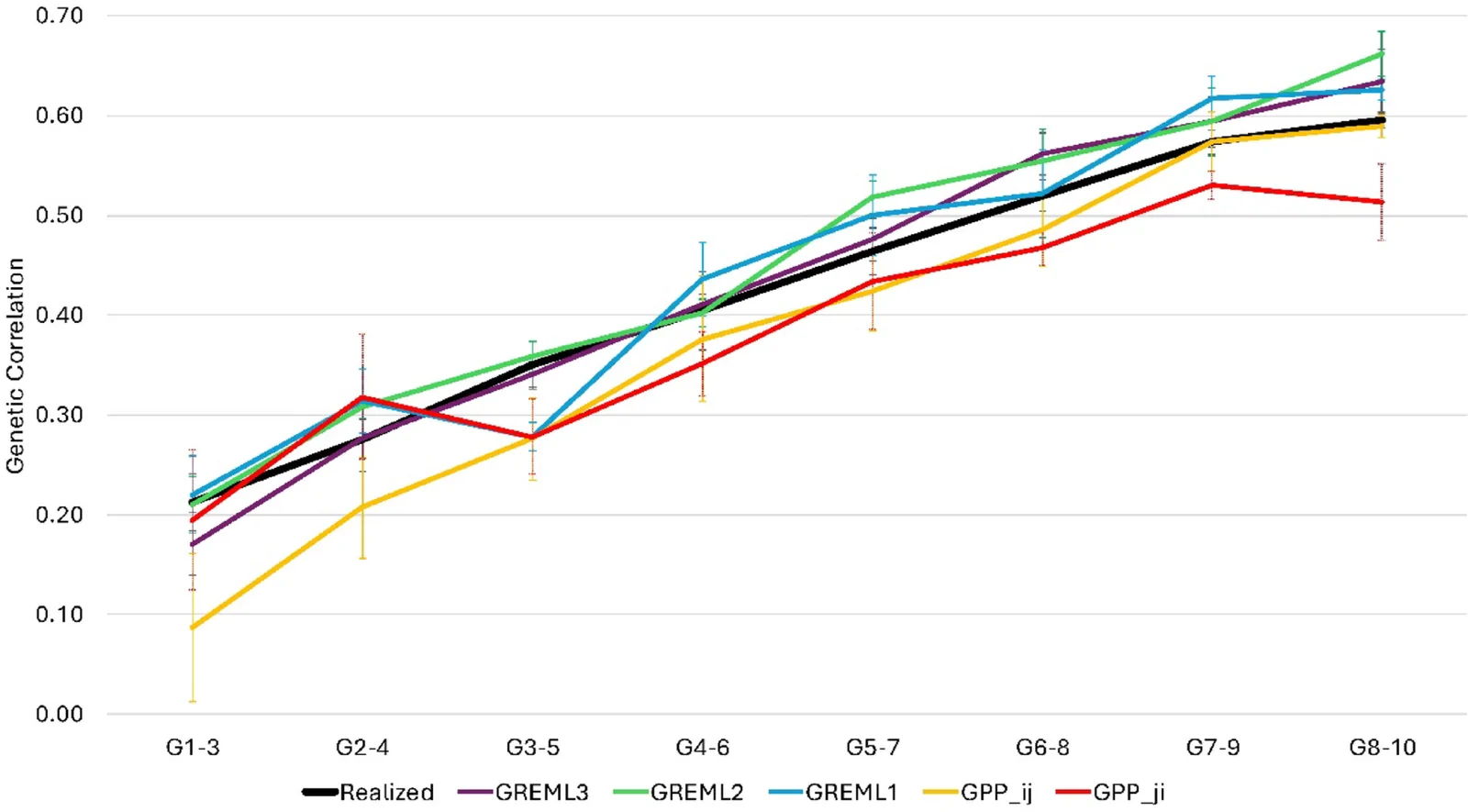
Realized and estimated genetic correlations between primary and secondary production traits over generations for the small dataset. Estimates are by Genomic REML (GREML) using 1 to 3 generations of phenotypes, and by genetic parameters via predictivity (GPP). The latter considered predictivity of production based on fitness (Gpp_ij_) and fitness based on production (Gpp_ji_). Standard errors (SE) are SE of replicates

Figure 8 shows genetic correlations between primary production and secondary production traits over generations for different methods. The realized genetic correlation increased from 0 in generation 1 to + 0.60 in generation 10. The genetic correlations estimated by GREML using a single generation were closer to the realized values than using more generations. Underestimation by GPP could be an artifact of inflated heritability for the secondary production trait obtained by GPP or underestimation due to the limited information for the low heritability trait. Again, as stated earlier, we hypothesize that such inflation will be reduced or eliminated when increasing the data size. The correlation strength (weak versus strong) did not affect the estimation of genetic correlations using GPP.

### GPP for large datasets

**Fig. 9:**
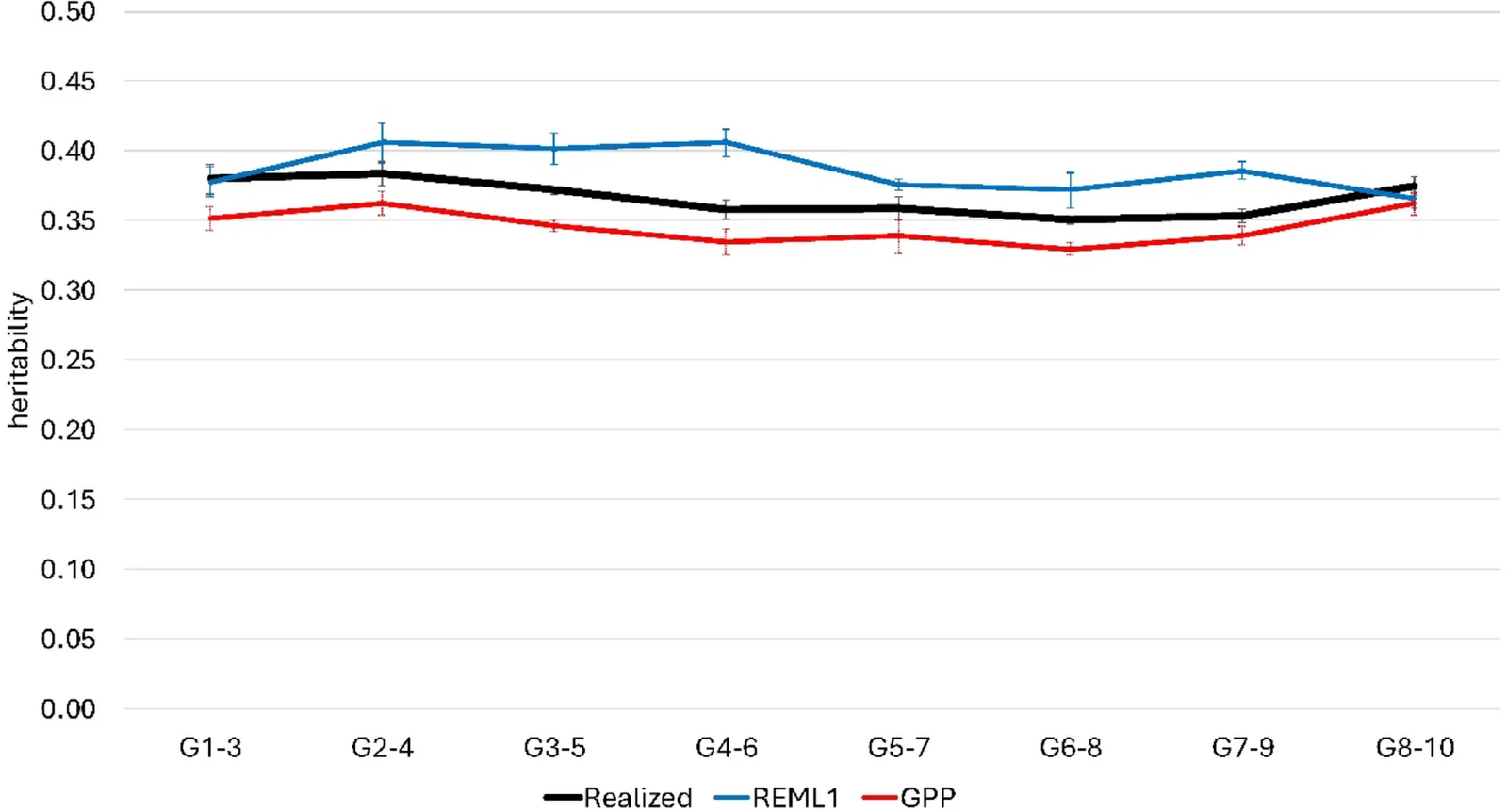
Realized and estimated heritabilities over generations for primary production trait in the large dataset. Estimates are genetic parameters via predictivity (GPP) and REML using one generation of phenotypes compared with realized values. Standard errors (SE) are SE of replicates

Realized and estimated heritabilities as a function of generation number for scenario B for the primary production trait are in Fig. 9. As the number of data points increased for estimating genetic parameters using GPP, the estimates were closer to the realized values. Estimates by GPP for a given generation used adjusted phenotypes from that generation, and GEBV obtained from observations of two previous generations. Due to computational limitations, GREML was not feasible. Thus, we used pedigree-based REML, which was only possible if applied to a single generation. Here, the selection pressure was more distributed across a much larger number of candidates, which led to slower loss of genetic variance due to more individuals contributing to the next generation, and better management of inbreeding. Larger populations retain more genetic variance, buffering the decline in heritability over generations. The estimates by GPP and REML closely followed the realized heritability. GPP slightly underestimated, and REML slightly overestimated the heritability.

**Fig. 10:**
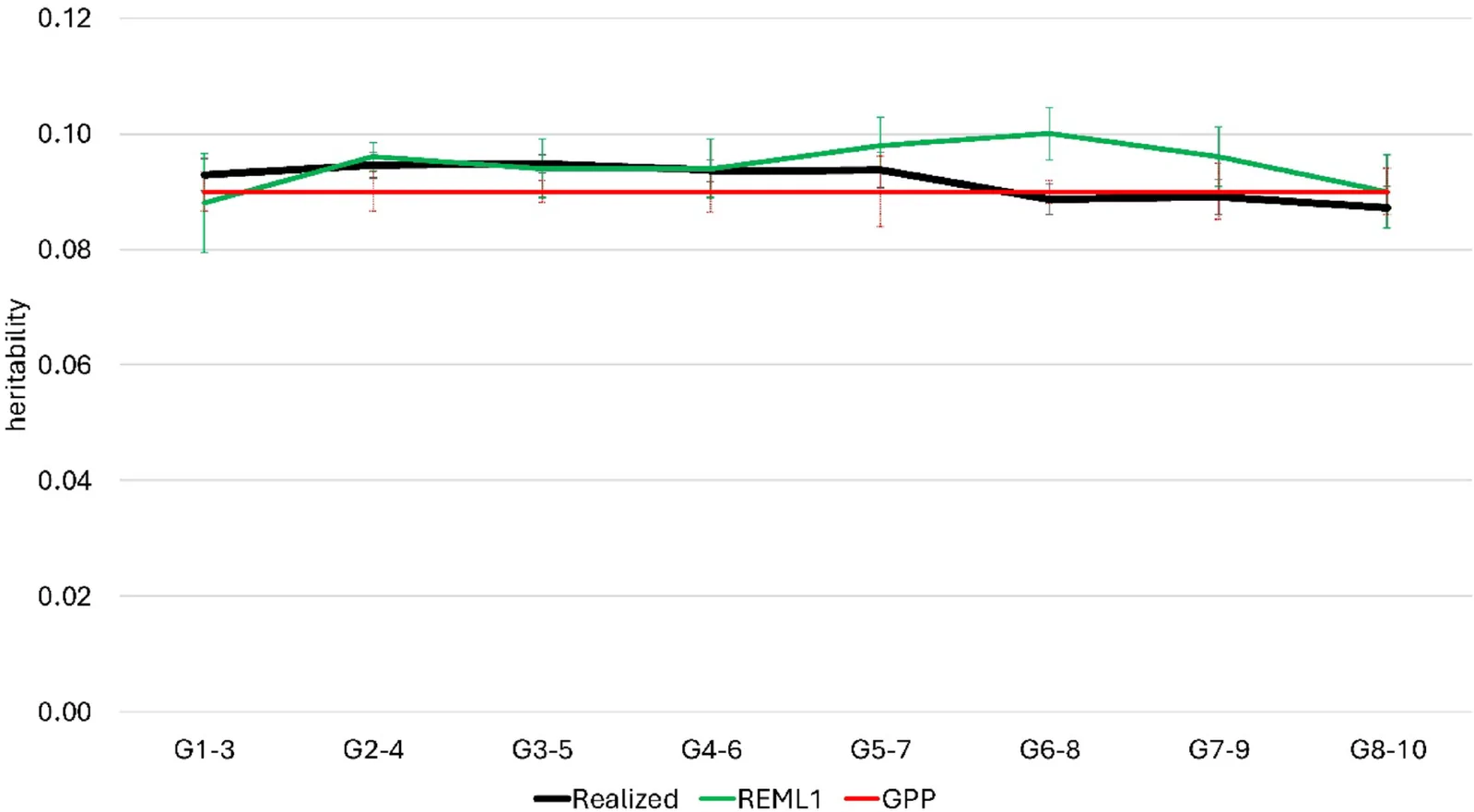
Realized and estimated heritabilities over generations for fitness in the large dataset. Estimates are genetic parameters via predictivity (GPP) and REML using one generation of phenotypes compared with realized values. Standard errors (SE) are SE of replicates

**Fig. 11:**
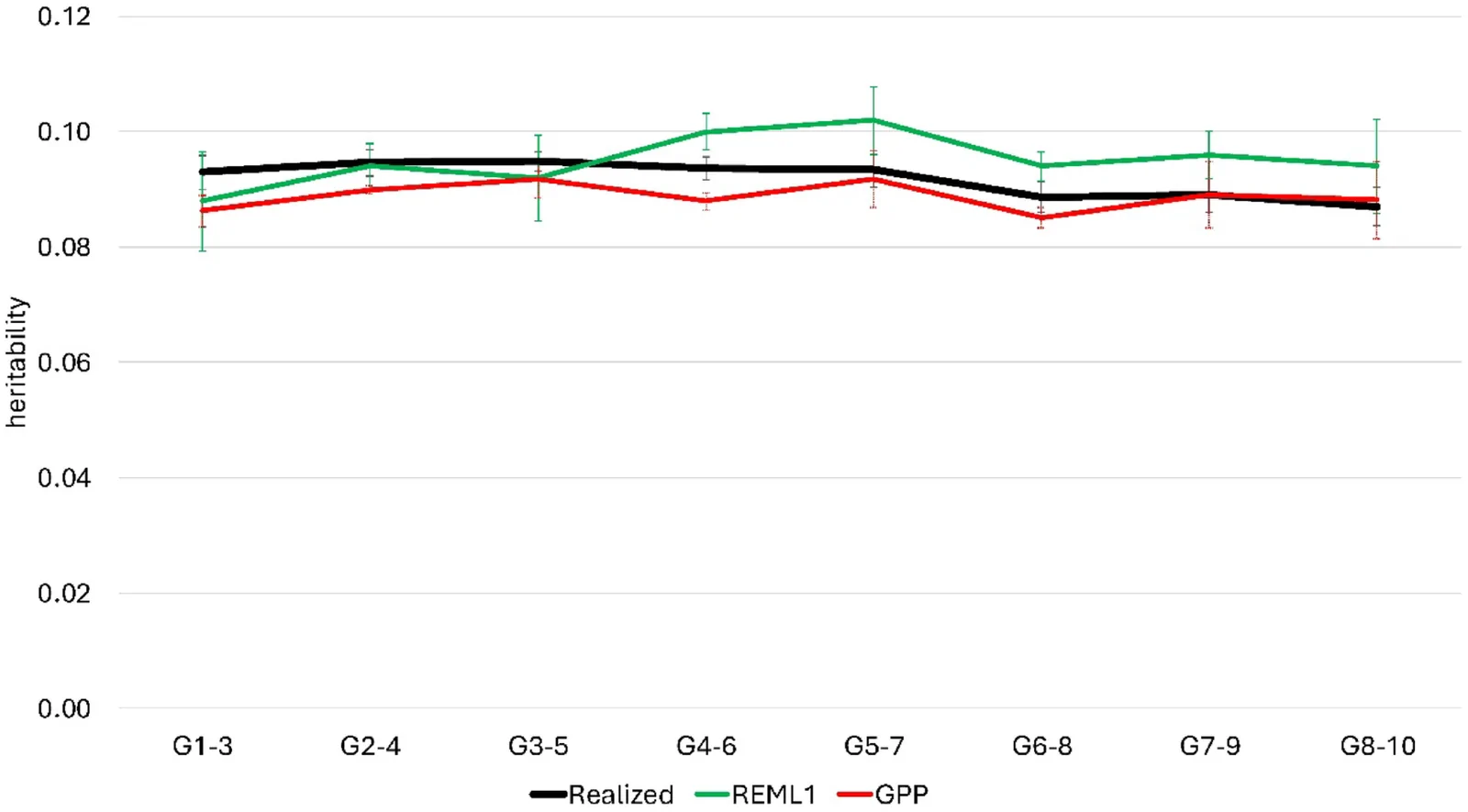
Realized and estimated heritabilities over generations for secondary production trait in the large dataset. Estimates are genetic parameters via predictivity (GPP) and REML using one generation of phenotypes compared with realized values. Standard errors (SE) are SE of replicates

Heritability estimates for the fitness trait and the secondary production trait are in Figs. 10 and 11, respectively. The heritability for these two traits was low; however, due to more data points, estimates by GPP closely followed the realized heritabilities for both traits. These two traits are composite traits obtained from a primary production trait, having either negative or positive correlations with the primary production trait. Due to an initial low heritability (0.10), a slight decline in heritability resulting from selection in the primary production trait was observed in both traits. REML estimates were close; however, inflation was noticed. While the primary production trait was under strong selection pressure for all generations, fitness and secondary production traits were indirectly selected.

**Fig. 12:**
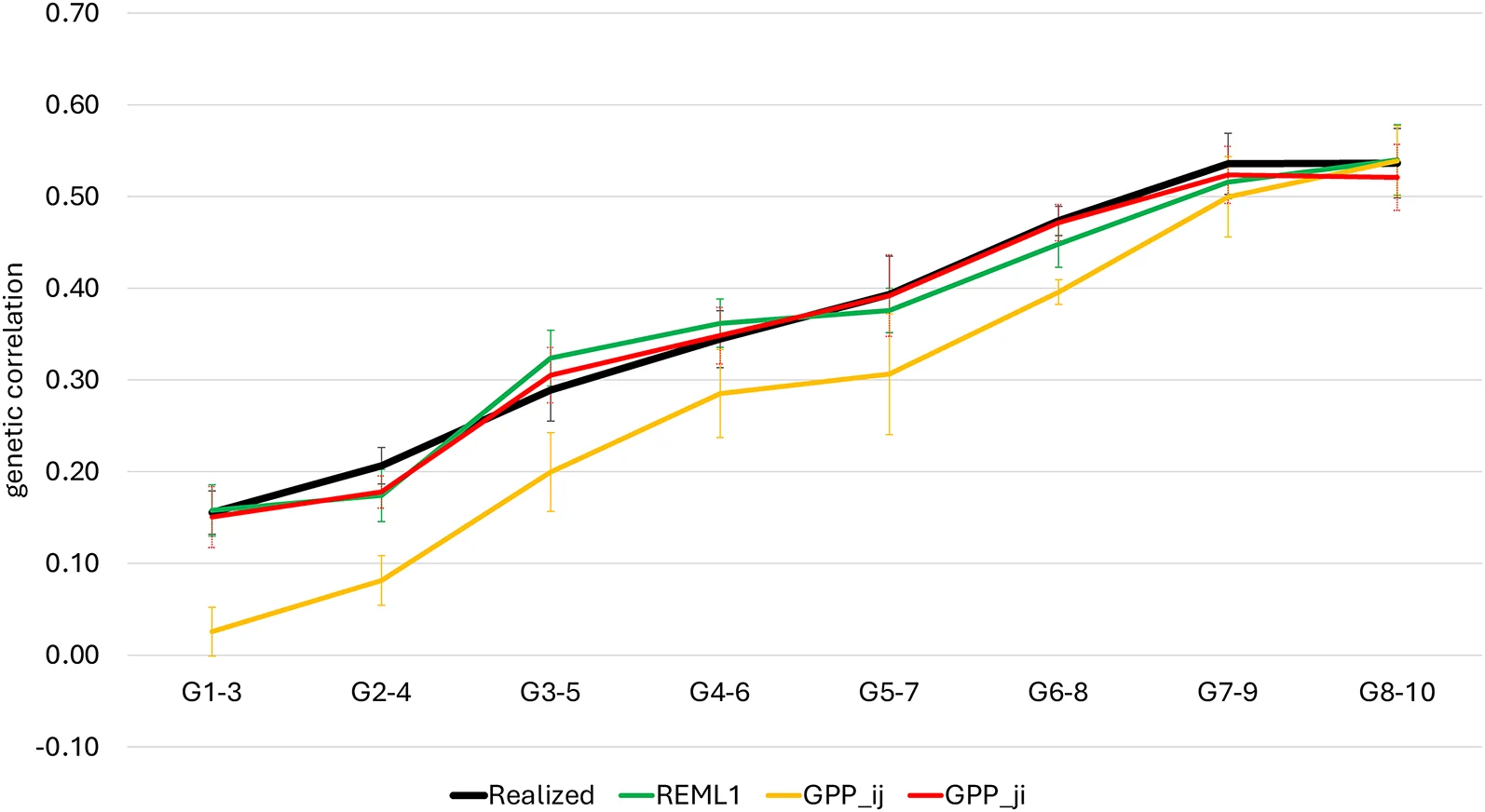
Realized and estimated genetic correlations between primary production and fitness traits over generations in the large dataset. Estimates are genetic parameters via predictivity (GPP) and REML using one generation of phenotypes compared with realized values. The GPP considered the predictivity of production based on fitness (Gpp_ij_) and fitness based on production (Gpp_ji_). Standard errors (SE) are SE of replicates

Genetic correlations between production and fitness are in Fig. 12. The realized genetic correlation declined from 0 in generation 1 to − 0.54 in generation 10. In the very large dataset, estimates by GPP were very close to the realized ones, whereas REML provided slightly underestimated values. In the smaller dataset, the genetic correlations estimated by GPP were closer to the realized ones when predicting adjusted phenotypes for fitness based on GEBV for production (GPP_ji_). In the large dataset, the same applies, and such estimates by GPP were very close to the realized ones. Therefore, GPP_ji_ were best as they were closer to the realized estimates than GPP_ij,_ which is predicting adjusted phenotypes for production based on GEBV for fitness. It was also seen that the estimates from GPP were close to realized estimates across the weak and strong negative genetic correlation scenarios. However, REML estimates were inflated for low correlation time slices and more accurate for strong correlation time slices. For GPP, the strength of the negative genetic correlation between the traits did not affect the estimates.

**Fig. 13:**
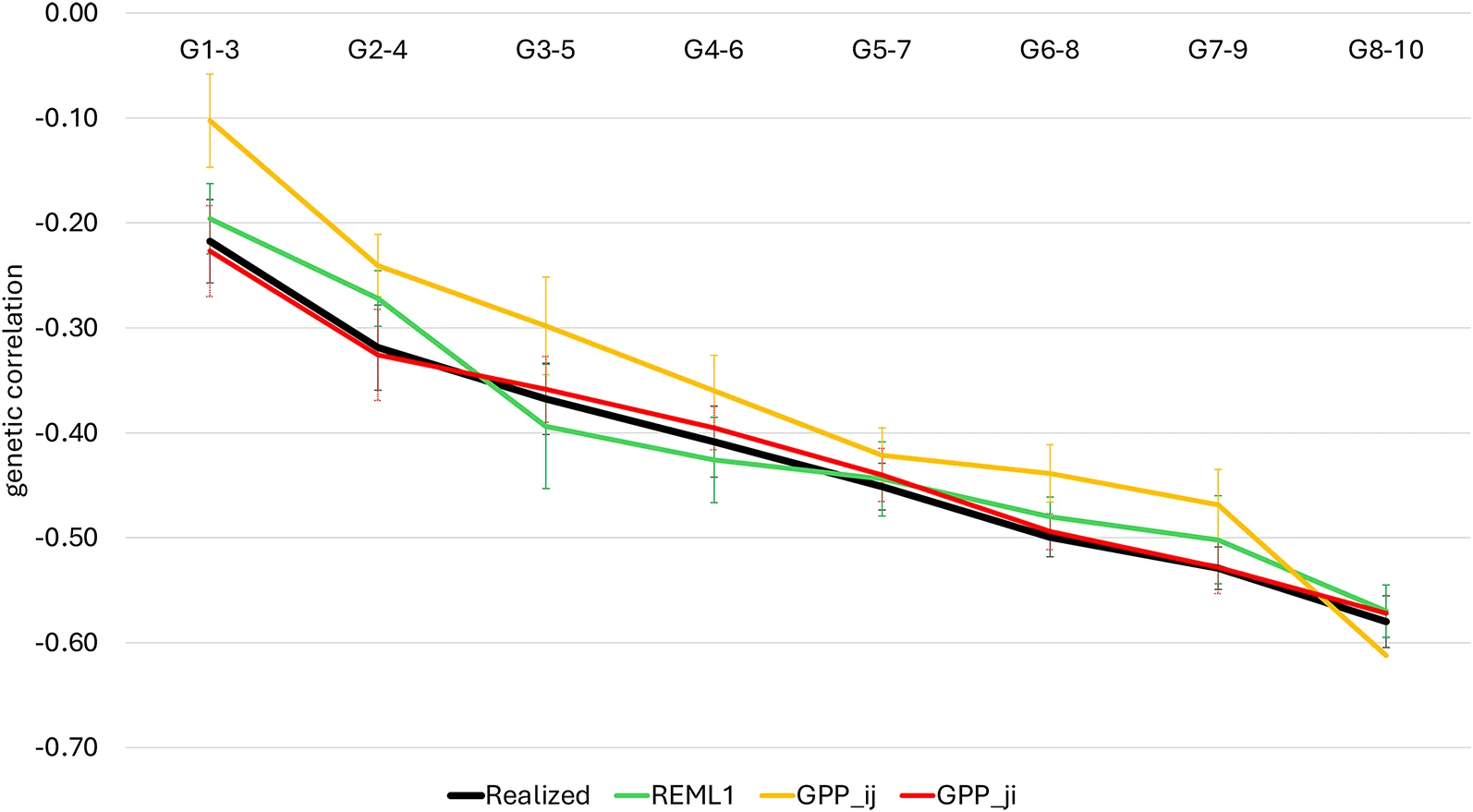
Realized and estimated genetic correlations between primary and secondary production traits over generations in the large dataset. Estimates are genetic parameters via predictivity (GPP) and REML using one generation of phenotypes compared with realized values. The GPP considered the predictivity of primary production based on secondary production trait (Gpp_ij_) and vice-versa (Gpp_ji_). Standard errors (SE) are SE of replicates

Figure 13 tracks the estimates of genetic correlations between primary and secondary production traits for the GPP and pedigree-based REML. The realized genetic correlation increased from 0 in generation 1 to + 0.51 in generation 10. Genetic correlations estimated by GPP were close to the realized estimates when predicting adjusted phenotypes for the secondary production trait based on GEBV for the primary production trait (GPP_ji_). Pedigree-based REML estimates were slightly overestimated, primarily at strong correlation time slices. The strength of the genetic correlation had no significant impact on the estimates by GPP. For weak (early time slices) and strong (later time slices) correlations, GPP_ji_ and realized estimates were very close.

### Overall GPP performance

In nearly all cases, the heritability and genetic correlation estimates followed the realized values. Notable exceptions were large fluctuations in pedigree-based REML for production in the small dataset and inflation by GPP for fitness in the small dataset. Large fluctuations in pedigree-based REML estimates were caused by the limited information available for estimation, although the estimates for fitness were generally reasonable. The inflation in GPP for small datasets makes this method non-applicable to populations of limited size, although the estimates by GPP for fitness and secondary production traits were reasonable. In the large dataset, trends for all estimates were similar to the realized values.

In this study, we observed that the focal group, for which estimates are desired, must have a number of individuals greater than or equal to the number of independent chromosome segments as defined by Stam [37], i.e., *M*_*e*_ = *4N*_*e*_*L*, to obtain reliable estimates using GPP. Having a number of animals in the focal group smaller than the expected *M*_*e*_ (*4N*_*e*_*L*) results in an inflated estimate of heritability (Additional file 2 Figure S1), further affecting the estimates of genetic correlations using GPP. In scenario A, eig98 estimates are lower than *4N*_*e*_*L* (Additional file 1 Table S1), and hence using actual eig98 estimates in place of *4N*_*e*_*L* in the GPP formula helps in reduction of bias for estimation of heritability. As for the upper limit, we have tested the method up to 100,000 individuals in a focal group and it worked well (results not shown for less than 100,000). We tested *M*_*e*_ = *4N*_*e*_*L* (6000) versus the actual eig98 estimate of 8355 to compute heritability for the primary production traits using GPP. The estimates were similar, indicating robustness of GPP for higher values of *M*_*e*_ in large populations (Additional File 2 Figure S2). Thus, we advocate that GPP can be used for large populations with many genotyped animals.

Reasonable genetic parameter estimates were obtained across all methods, indicating consistency among the evaluated approaches, except pedigree-based REML with only one generation of data. Cesarani et al. [21] also reported large biases in estimates of variance components in pedigree-based REML analyses with one generation of data. Selective genotyping can bias estimates of genetic parameters, as shown for GREML estimates [21]; however, all individuals were genotyped in our study. Genetic parameters for fitness may have been affected by the complex structure of this trait and by the less-than-optimal model of analysis, given its composite nature. The changing nature of the fitness trait may pose challenges when many generations are analyzed simultaneously, but less so when fewer generations are considered. Based on the observed change in genetic correlation across generations in our results, a change of approximately 0.1 per generation would imply a bias of similar magnitude when three generations are analyzed jointly.

The GPP estimates from the large dataset (scenario B) were very close to the realized values. This is an important feature of GPP, given that analyses using large genomic data in REML-based methods are not feasible. Genomic GIBBS has a slightly higher capacity, but a significantly higher computational cost. With the large dataset, GPP was obtained in 55 min, an efficiency that could never be obtained with other methods, even if they were feasible. In general, the computational cost and memory requirements of GPP are approximately linear with the number of genotyped individuals, whereas those of genomic GIBBS are approximately quadratic.

Genetic correlation estimates were obtained using GPP and were compared with the realized estimates to examine their dynamics under strong and weak correlation scenarios. The genetic correlations estimated by GPP were closer to the realized ones when predicting adjusted phenotypes for fitness based on GEBV for production (GPPji), but not for GPP_ij_ across all the scenarios. We observed that the strength and direction of the genetic correlation was accurately captured by the GPP estimates. Across earlier time slices (weaker correlation) and later time slices (stronger correlation), the GPP estimates of genetic correlation (positive or negative) were fairly similar to the realized estimates in large datasets. In real populations, temporal changes in genetic correlations may result from allele-frequency shifts caused by selection, changes in SNP effects (e.g., genotype-by-time interactions), or both; disentangling these components was beyond the scope of the present simulation study.

Estimates by GPP can be computed whenever predictivities can be computed. The formula for predictivity includes phenotypes adjusted for fixed effects [19]. In complex models with several random effects, some of them may be correlated. In practice, adjustments included all fixed and random effects, except the one being predicted (e.g., the animal effect, **u**). However, it is not clear how to calculate predictivity in models that include maternal effects or random regressions, which deserves further investigation.

It is important to note that the simulation was specifically for GBLUP since all phenotyped animals were also genotyped. In animal populations, only a fraction of animals are genotyped, and genomic evaluations are mainly by ssGBLUP; however, the equation provided in [23] applies to GBLUP only. When the number of genotyped animals was large, GPP [22] found a good agreement between the prediction accuracy equations provided in [23] and [19] when *N* was the number of individuals with both genotypes and phenotypes. Nonetheless, for a large *N*, GPP based on ssGBLUP and GBLUP may converge to the same point.

## Conclusions

We evaluated the robustness and applicability of a new method for estimating heritability and genetic correlation based on predictivities, called Genetic Parameters via Predictivity (GPP). The method offers a fast, flexible, and accurate approach for estimating dynamic genetic parameters across time slices in large datasets subject to genomic evaluations when predictivity is feasible. The GPP feature of estimating parameters based on time slices is especially desirable to reflect estimates in the validation population. With that, investigating the dynamics of heritability and genetic correlations over time becomes feasible for a large genotyped population, where traditional methods like REML and GIBBS are ruled out. We observed that the GPP method performs better when the focal group has a number of animals that is equal to or larger than the number of independent chromosome segments. Below that, the genetic parameter estimates can be inflated. Overall, GPP performs well across a wide range of genetic correlations, from weak to strong, whether positive or negative. These results suggest that GPP is a robust and scalable method for estimating dynamic genetic parameters in large populations under genomic selection. Validating GPP to real datasets may provide further insight into its robustness and practical relevance in breeding programs.

## Electronic Supplementary Material

Below is the link to the electronic supplementary material.

Supplementary Material 1

Supplementary Material 2

## Data Availability

The datasets used in this study were simulated and are available upon request.
