# Correction: Genetic parameters via predictivity in large populations under strong genomic selection

**DOI:** 10.1186/s12711-026-01089-y

**Published:** 2026-09-23

**Authors:** Gopal Gowane, Jorge Hidalgo, Mary Kate Hollifield, Ignacy Misztal, Daniela Lourenco

**Affiliations:** 1 Department of Animal and Dairy Science, University of Georgia, Athens, GA 30602 USA; 2 Animal Genetics and Breeding Division, ICAR-National Dairy Research Institute, Karnal, 132001 India

**Correction: Genet Sel Evol (2026) 58:39**

**https://doi.org/10.1186/s12711-026-01054-9**

Following publication of the original article [1], the authors identified an error in Fig. 12 and Fig. 13. The title for the y-axis in these figures should read as ‘genetic correlation’ in place of ‘heritability’.

The incorrect and the correct figure is given below.

The incorrect Fig. 12

**Figure:**
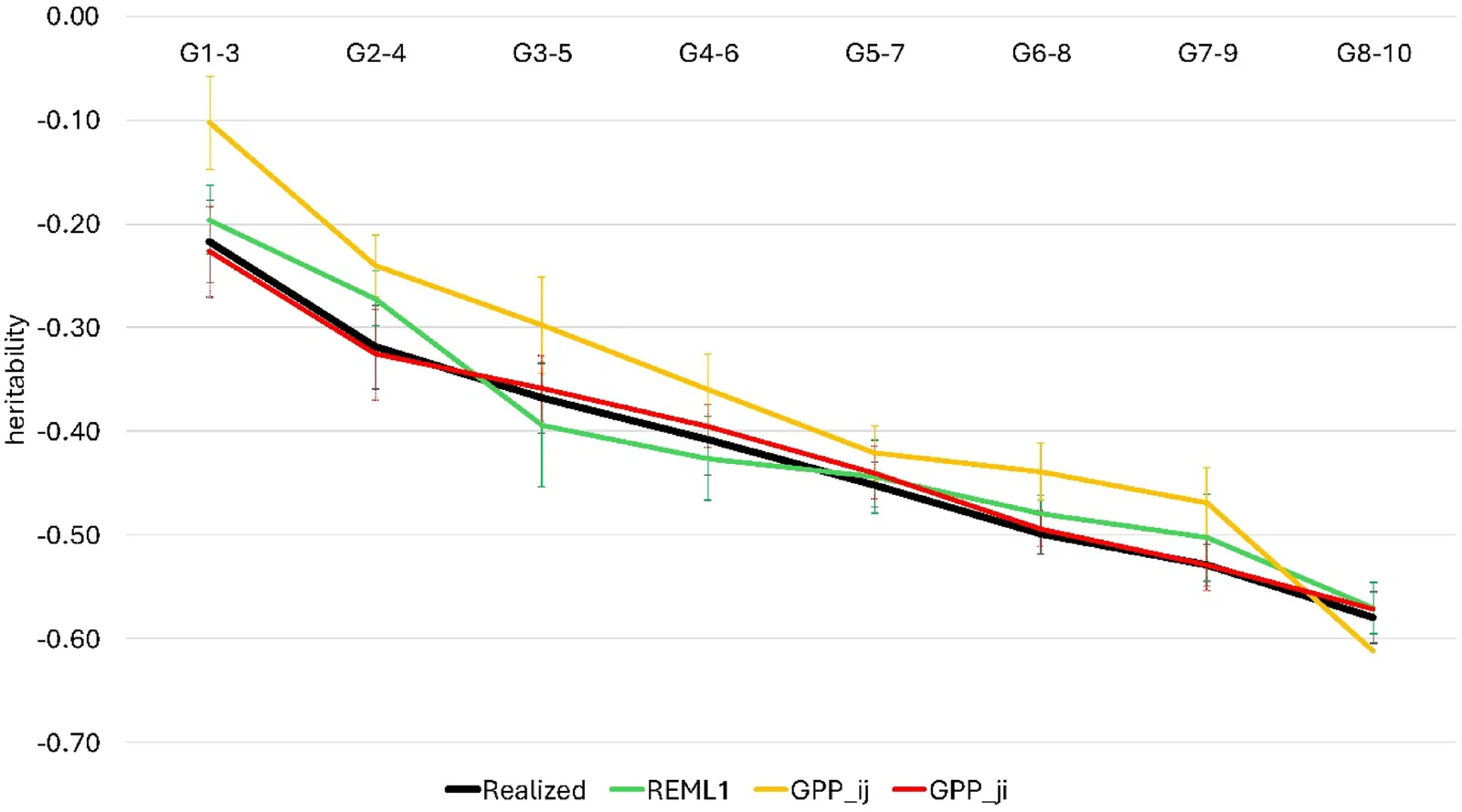

The correct Fig. 12

**Figure:**
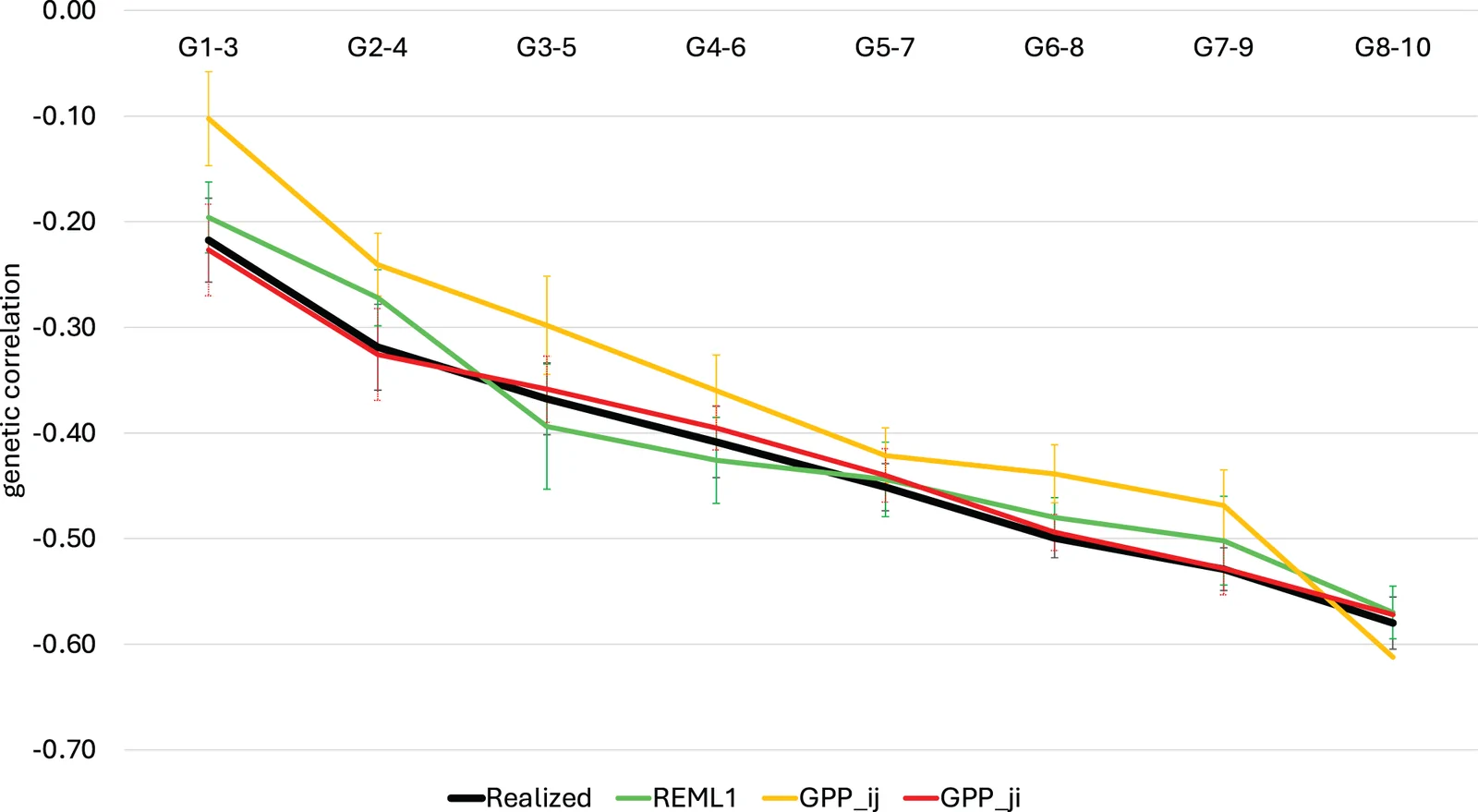

The incorrect Fig. 13

**Figure:**
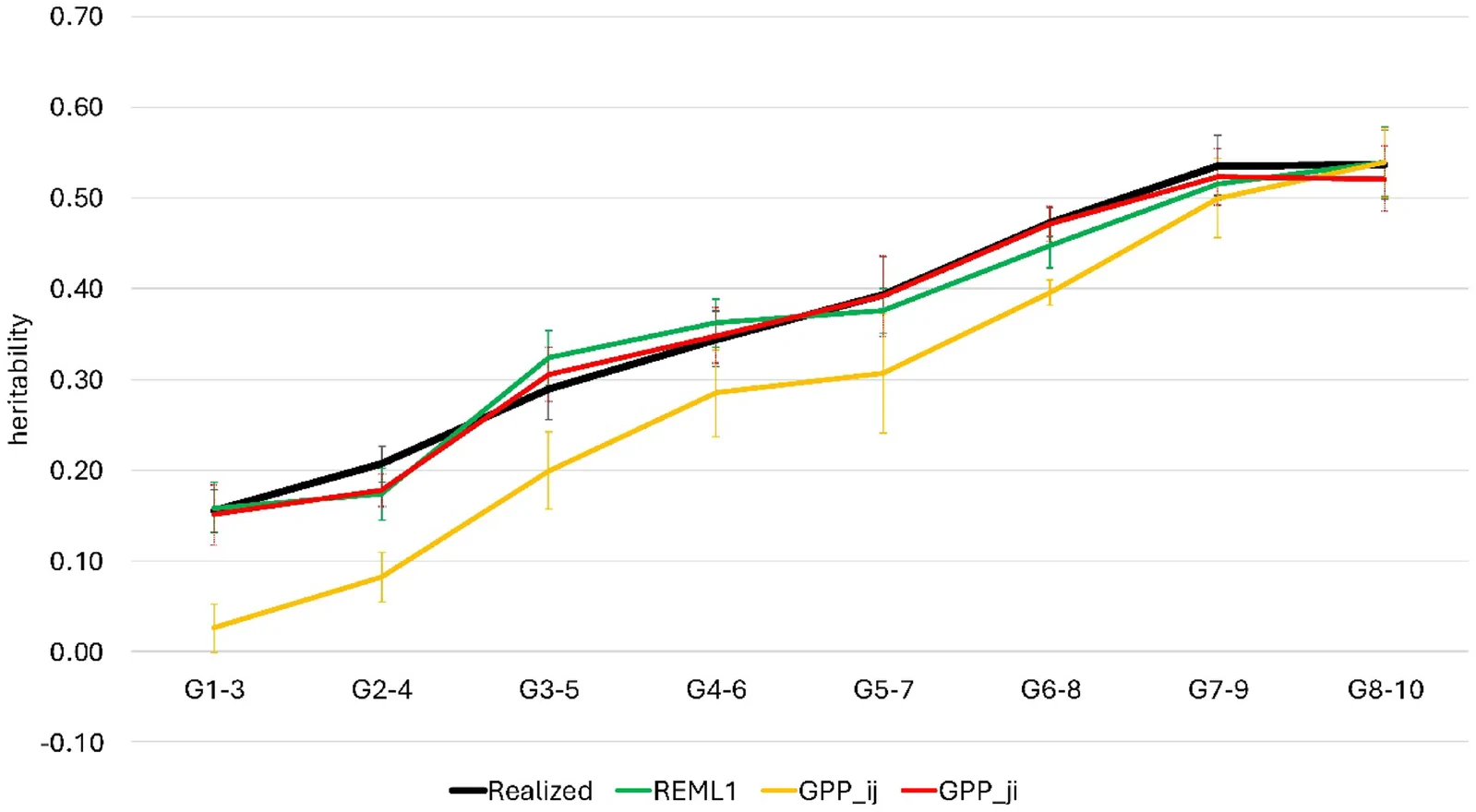

The correct Fig. 13

**Figure:**
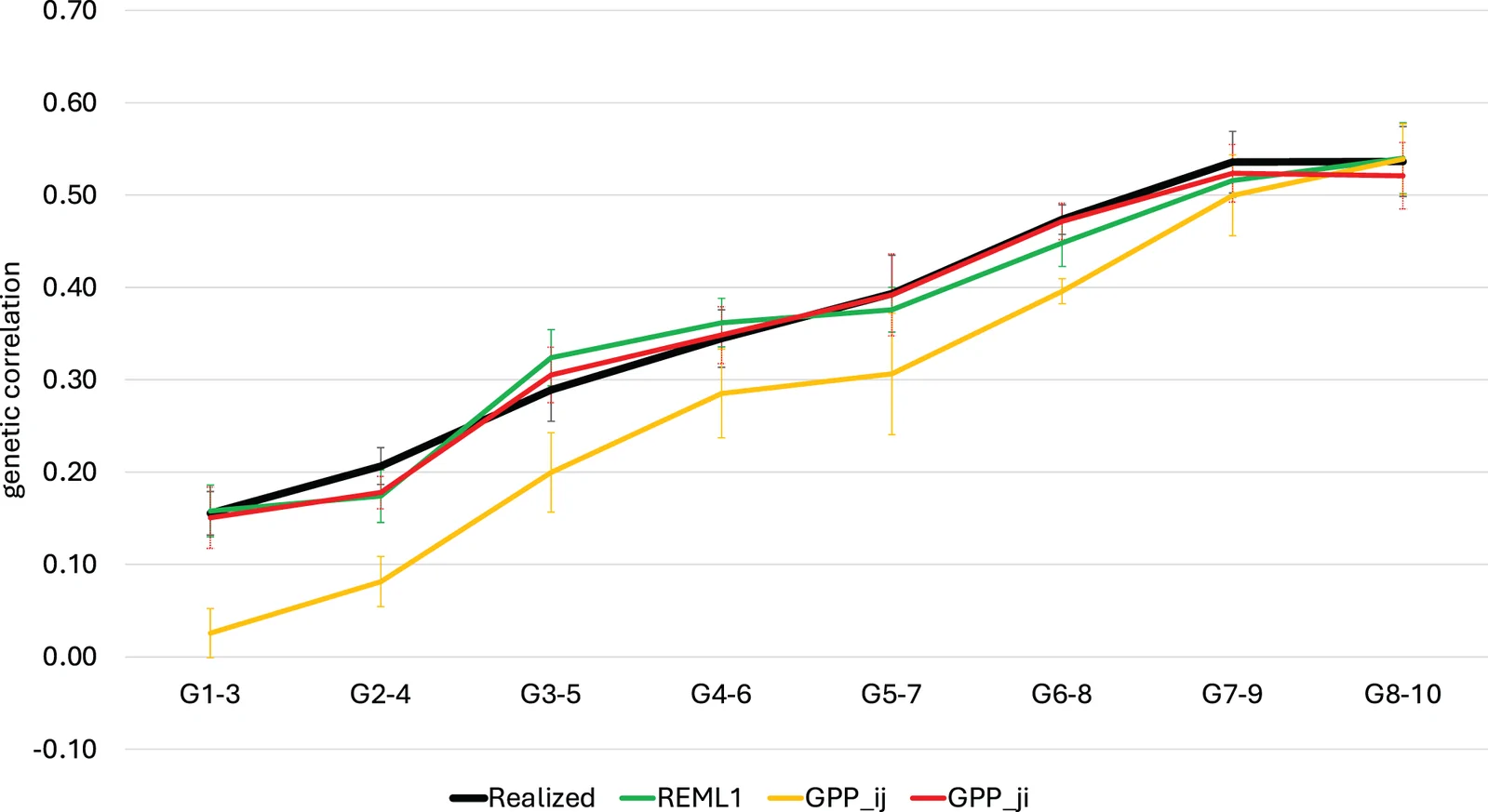

The original article [1] has been corrected.
