# Supplementary material for: Genetic parameters via predictivity in large populations under strong genomic selection
Source: Genet Sel Evol. 2026 Jun 7;58:39. doi: 10.1186/s12711-026-01054-9 (PMC13463401; doi:10.1186/s12711-026-01054-9)
Supplement: Supplementary file 1 [file 12711_2026_1054_MOESM1_ESM.docx]

**Table S1. Change in the population structure for every generation due to selection**

| Period | Scenario A | | Scenario B | |
| --- | --- | --- | --- | --- |
|  | eig99 | eig98 | eig99 | eig98 |
| Gen1 | 4007.00±1.20 | 3475.70±2.85 | 11931.00±30.00 | 8801.00±4.00 |
| Gen2 | 4000.70±0.70 | 3473.30±2.33 | 12355.50±11.50 | 9106.00±13.00 |
| Gen3 | 3963.70±3.20 | 3418.70±5.55 | 12516.00±5.00 | 9177.00±17.00 |
| Gen4 | 3908.00±11.10 | 3348.00±14.19 | 12379.50±28.50 | 9000.50±17.50 |
| Gen5 | 3848.00±20.00 | 3272.30±26.36 | 12098.00±52.00 | 8724.50±39.50 |
| Gen6 | 3781.00±28.00 | 3193.30±34.38 | 11769.00±84.00 | 8420.00±66.00 |
| Gen7 | 3706.00±40.00 | 3108.00±46.76 | 11342.50±99.50 | 8047.50±83.50 |
| Gen8 | 3629.70±54.70 | 3024.67±61.37 | 10954.00±107.00 | 7724.00±93.00 |
| Gen9 | 3568.70±56.70 | 2954.33±62.72 | 10580.50±117.50 | 7408.00±100.00 |
| Gen10 | 3510.30±59.30 | 2888.67±65.40 | 10241.00±113.00 | 7142.00±97.00 |

*eig98: number of largest eigenvalues explaining 98% (eig98) of the variance in genomic relationship matrix*

*eig99: number of largest eigenvalues explaining 99% (eig99) of the variance in genomic relationship matrix*
