# Supplementary material for: Genetic parameters via predictivity in large populations under strong genomic selection
Source: Genet Sel Evol. 2026 Jun 7;58:39. doi: 10.1186/s12711-026-01054-9 (PMC13463401; doi:10.1186/s12711-026-01054-9)
Supplement: Supplementary file 2 [file 12711_2026_1054_MOESM2_ESM.docx]

Heritability

|  | GPP_4NeL | GPP_eig98 | Realized |
| --- | --- | --- | --- |
| G1-3 | 0.418262 | 0.345117 | 0.349955 |
| G2-4 | 0.412591 | 0.337095 | 0.333237 |
| G3-5 | 0.399699 | 0.322633 | 0.320207 |
| G4-6 | 0.408796 | 0.328541 | 0.328567 |
| G5-7 | 0.40986 | 0.326397 | 0.324693 |
| G6-8 | 0.399518 | 0.314289 | 0.314695 |
| G7-9 | 0.395106 | 0.307677 | 0.303203 |
| G8-10 | 0.388677 | 0.30059 | 0.303676 |

**Figure S1. Estimates for heritability of primary production trait in small data scenario (A) for expected versus estimated *M_e_***

*GPP_4NeL: estimate for heritability using GPP with M_e_ as 6000 (4N_e_L)*

*GPP_eig98: estimate for heritability using GPP with M_e_ as eig98 in focal generation as mentioned in Supplementary Table1*

*Realized: estimate for heritability as observed in data*

Heritability

|  | GPP_4NeL | GPP_eig98 | Realized |
| --- | --- | --- | --- |
| G1-3 | 0.343032 | 0.343032 | 0.372201 |
| G2-4 | 0.354055 | 0.354055 | 0.379689 |
| G3-5 | 0.343382 | 0.343382 | 0.369409 |
| G4-6 | 0.341004 | 0.341004 | 0.3622 |
| G5-7 | 0.335029 | 0.335029 | 0.359432 |
| G6-8 | 0.329326 | 0.329326 | 0.347057 |
| G7-9 | 0.33872 | 0.33872 | 0.354359 |
| G8-10 | 0.359982 | 0.359982 | 0.373821 |

**Figure S2. Estimates for heritability of primary production trait in large data scenario (B) for expected versus estimated *M_e_***

*GPP_4NeL: estimate for heritability using GPP with M_e_ as 6000 (4N_e_L)*

*GPP_eig98: estimate for heritability using GPP with M_e_ as eig98 in focal generation as mentioned in Supplementary Table1*

*Realized: estimate for heritability as observed in data.*
